# Experimental activation assessment of open‐type yoke in a PET cyclotron by non‐destructive/destructive γ‐ray measurement approaches

**DOI:** 10.1002/mp.70649

**Published:** 2026-08-25

**Authors:** Go Yoshida, Hiroshi Matsumura, Akihiro Toyoda, Hajime Nakamura, Kazuyoshi Masumoto, Taichi Miura, Eisuke Watanabe, Kiyokazu Tsugane, Katsuhiko Saito, Iwane Koumura, Hiroyuki Uno, Masahiko Kozaki, Ayumi Yamamoto, Jun Toyohara, Kei Wagatsuma

**Affiliations:** ^1^ High Energy Accelerator Research Organization (KEK) Tsukuba Ibaraki Japan; ^2^ Sumitomo Heavy Industries, Ltd. Tokyo, Shinagawa‐ku Japan; ^3^ SHI‐ATEX Co., Ltd. Saijo Ehime Japan; ^4^ Tokyo Metropolitan Institute for Geriatrics and Gerontology Tokyo, Itabashi‐ku Japan; ^5^ Hokkaido University Sapporo Hokkaido Japan

**Keywords:** activation, cyclotron, non‐destructive measurement, positron emission tomography, radioactive waste

## Abstract

**Background:**

During decommissioning of positron emission tomography (PET) cyclotron facilities, metallic components—including the iron yoke—are typically classified as radioactive waste, incurring substantial disposal costs. In contrast to concrete, standardized decommissioning procedures for metallic components have not yet been established.

**Purpose:**

This study aimed to experimentally assess activation in a PET cyclotron and provide fundamental data for establishing decommissioning guidelines for metallic components. The investigation focused on residual activity in the yoke and center pole, which are among the heaviest structural parts.

**Methods:**

A PET cyclotron that operated for 23 years at the Tokyo Metropolitan Institute for Geriatrics and Gerontology was examined 6 years after the cyclotron's complete shutdown using two complementary approaches: non‐destructive γ‐ray measurements and destructive core‐boring analysis.

**Results:**

^60^Co was the predominant residual radionuclide. On the outer surfaces, ^60^Co specific activity typically ranged from 0.02 to 0.10 Bq/g, with many locations below the 0.1 Bq/g clearance level. Cross‐sectional mapping showed exponential attenuation behavior from the interior outward. Higher activation in the center pole (up to ∼1.0 Bq/g) was observed. ^54^Mn was one to two orders of magnitude lower than ^60^Co at 6 years post‐shutdown. Non‐destructive and destructive results were in close agreement, validating the quantitative use of γ‐detectors for near‐surface activity. A hypothetical time‐dependent decay estimation, based on the non‐destructive/destructive findings, was performed to project a required cooling period to meet clearance.

**Conclusions:**

Activation levels in PET cyclotron components can be effectively assessed using combined non‐destructive and destructive approaches. The complex distribution of residual activity was observed in the yoke and center poles. Decay projections indicated that approximately half of the yoke would fall below the clearance level within 15 years and nearly all components within 23 years after shutdown. These findings provide essential benchmarks for improving simulation‐based activation modeling and can be broadly applied to similar PET cyclotron systems.

## 1 INTRODUCTION

Positron emission tomography (PET) is widely utilized for cancer diagnosis across Japan. This accessibility is supported by more than 150 cyclotrons dedicated to the production of PET radiopharmaceuticals (PET cyclotrons).^1^ However, many of these PET cyclotrons are approaching the end of their operational lifespans and are expected to be decommissioned in the near future. Therefore, accurate assessments of activation levels and residual radionuclides in both the concrete of the accelerator room and the metallic components of the cyclotron—together comprising most of a PET cyclotron facility's mass—are essential to ensure safe and cost‐effective disposal.

The experimental assessment procedures for residual activity in concrete—used extensively in building structures and representing the bulk of decommissioning waste—have been established,^2^, ^3^, ^4^, ^5^, ^6^, ^7^, ^8^, ^9^, ^10^ whereas standardized procedures for the cyclotron main body, which can weigh tens of tons, have not. Among its components, the yoke, which was made from pure iron, accounts for a substantial portion of the mass and presents a potential burden in terms of radioactive waste management. Therefore, this study focuses on the experimental assessment of practical techniques in an actual PET cyclotron facility for measuring residual activity within the yoke, discussing both surface and internal activity distributions.

Activation of the yoke arises from exposure to secondary neutrons generated by reactions between protons—the accelerated particles—and constituent atoms. As PET cyclotrons typically operate at energies below 20 MeV, the dominant activation mechanisms involve proton‐induced (p,n) reactions and neutron‐induced (n,p) and (n,γ) reactions.^11^, ^12^ From a waste‐management perspective, the radionuclides of particular concern are ^54^Mn (half‐life: 312 days^13^), produced from iron—the main constituent of the yoke—and ^60^Co (half‐life: 5.27 years^13^), produced from cobalt impurities in the iron. A study using core boring from the yoke indicates that the dominant induced nuclides are ^54^Mn and ^60^Co,^11^ and that ^60^Co is also the dominant induced activity in the coil.^12^

To determine the distribution of residual activity on the yoke surface, a rapid and straightforward non‐destructive screening method using γ‐ray detectors—previously validated for assessing concrete activity in PET cyclotron facilities—was adopted, and its applicability to the yoke was investigated. This method employs two types of detectors: a cesium iodide (CsI) scintillation survey meter and a cerium bromide (CeBr_3_) scintillation spectrometer. Both were enclosed in custom lead shields to minimize background interference during surface scanning. Since the detectors provide different outputs—contact dose rate (CsI) and γ‐ray spectra (CeBr_3_)—a calibration experiment was conducted prior to the assessment to establish a conversion between these outputs. The calibrated detectors were then used to map residual activity on the outer surface of the yoke.

As the yoke is a bulk iron component, it is also essential to determine internal activity for comprehensive waste assessment. While the non‐destructive approach is fast and convenient, it yields surface‐biased information. Therefore, a more precise but labor‐intensive destructive approach—direct sampling by core boring—was performed at four representative locations selected from the non‐destructive surface survey. The extracted cores were segmented and analyzed via γ‐ray spectrometry using a high‐purity germanium (Ge) detector to derive depth profiles of residual activity. These depth‐resolved data provide direct evidence of how residual activity extends into the interior of the yoke, enabling accurate estimation of the total inventory. Moreover, the destructive approach is indispensable for validating non‐destructive measurements and for determining whether the component can meet clearance criteria after an appropriate cooling period. By complementarily employing non‐destructive and destructive approaches with differing advantages, we establish a practical and efficient decommissioning assessment strategy that balances feasibility and accuracy through a synergistic effect.

Since PET cyclotron usage is relatively uniform across facilities, the findings from this study are expected to be applicable to other installations. In addition, although international decommissioning guidelines (e.g., from the International Atomic Energy Agency [IAEA] or the U.S. Department of Energy [DOE]) exist, they often focus on nuclear reactors or large accelerators.^14^, ^15^ Our work addresses a gap in practical assessment approaches for smaller medical cyclotrons, which are increasingly used worldwide.

## 2 METHODS

### 2.1 Target machine

Cyclotrons for PET radiopharmaceutical production can be categorized by accelerated particle charge (positive‐ion or negative‐ion cyclotrons) and yoke geometry; representative models are summarized in Table 1.^16^, ^17^, ^18^, ^19^ First, with respect to particle charge, positive‐ion cyclotrons accelerate protons (including deuterons), and negative‐ion cyclotrons accelerate negatively charged hydrogen ions (including deuterium ions). In the positive‐ion cyclotrons, a deflector alters the beam trajectory and directs it toward the radioisotope (RI) production target. In the negative‐ion cyclotrons, ions are stripped of their electrons by a carbon stripper foil, converted to protons, and then directed to the target. Second, by yoke geometry, cyclotrons are classified as “open‐type” or “closed‐type” (Figure S1). The former comprises top and bottom horizontal yokes and two side vertical yokes, forming a rectangular frame with a hollow central section. Within this central region, the center poles, coils, and dees are installed, with a vacuum chamber positioned between the top and bottom yokes. In the latter, the vacuum chamber, coils, and dees are enclosed by a cylindrical yoke and are not visible externally. In both types, the yokes and center poles are made of high‐purity iron and constitute most of the main‐body mass.

**TABLE 1:** Classification of PET cyclotrons by particle charge and yoke geometry, with representative models and specifications.

Product name	Cypris370^16^ (This work)	Cypris HM‐18^17^	BC‐1710 ^18^	Cyclone 10/5^19^
Manufacturer	Sumitomo Heavy Industries, Ltd.	Sumitomo Heavy Industries, Ltd.	Japan steel Works, Ltd.	Ion Beam Applications SA
Yoke geometry ^a^	Open‐type	Open‐type	Closed‐type	Closed‐type
Particle charge	Positive‐ion	Negative‐ion	Positive‐ion	Negative‐ion
Maximum Energy (MeV)	18 (p), 10 (d)	18 (p), 10 (d)	17 (p), 10 (d)	10 (p), 5 (d)
Maximum current (µA) ^b^	50 (p), 10 (d)	70 (p), 50(d)	50	80 (p), 40 (d)

^a^Representative images are shown in Figure S1.

^b^Internal beam current.

**FIGURE 1:**
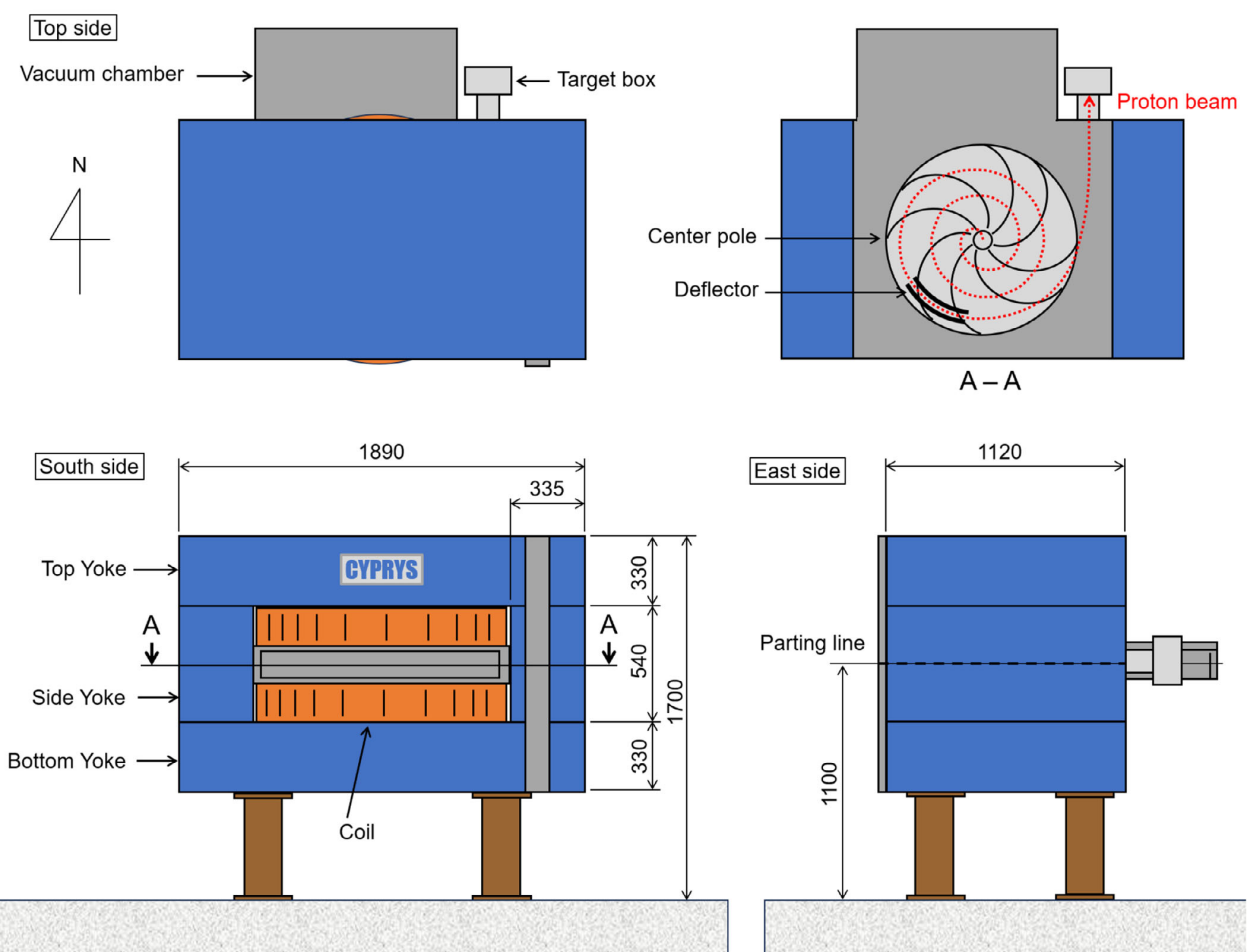
Schematic drawing of the PET cyclotron Cypris370 (unit: mm). The side yokes can be separated along the parting line corresponding to the beam acceleration plane, allowing the upper half to be lifted by 500 mm. The vacuum chamber was removed during the experiments.

The cyclotron investigated here, Cypris370^16^ (Sumitomo Heavy Industries, Ltd.), belongs to the positive‐ion and Open‐Type Category. Figure 1 shows the overview. The yokes and vacuum chamber can be separated at the beam acceleration plane, allowing the upper half of the yoke to be lifted by 500 mm. Protons are accelerated within the vacuum chamber in a horizontal orbit. Two major neutron sources are present: the RI production target and the deflector made from C1020 grade of oxygen‐free‐copper used to extract the proton beam. The machine is designed to accelerate protons up to 18 MeV and deuterons up to 10 MeV, with maximum internal beam currents of 50 µA (protons) and 10 µA (deuterons). It operated for 23 years (March 1990–March 2013) at the Tokyo Metropolitan Institute of Gerontology (reorganized as the “Tokyo Metropolitan Institute for Geriatrics and Gerontology” in 2009),^20^ with an average annual operation time of ∼200 h.

### 2.2 Non‐destructive activation assessment using γ‐ray detectors

#### 2.2.1 Experimental setups

**FIGURE 2:**
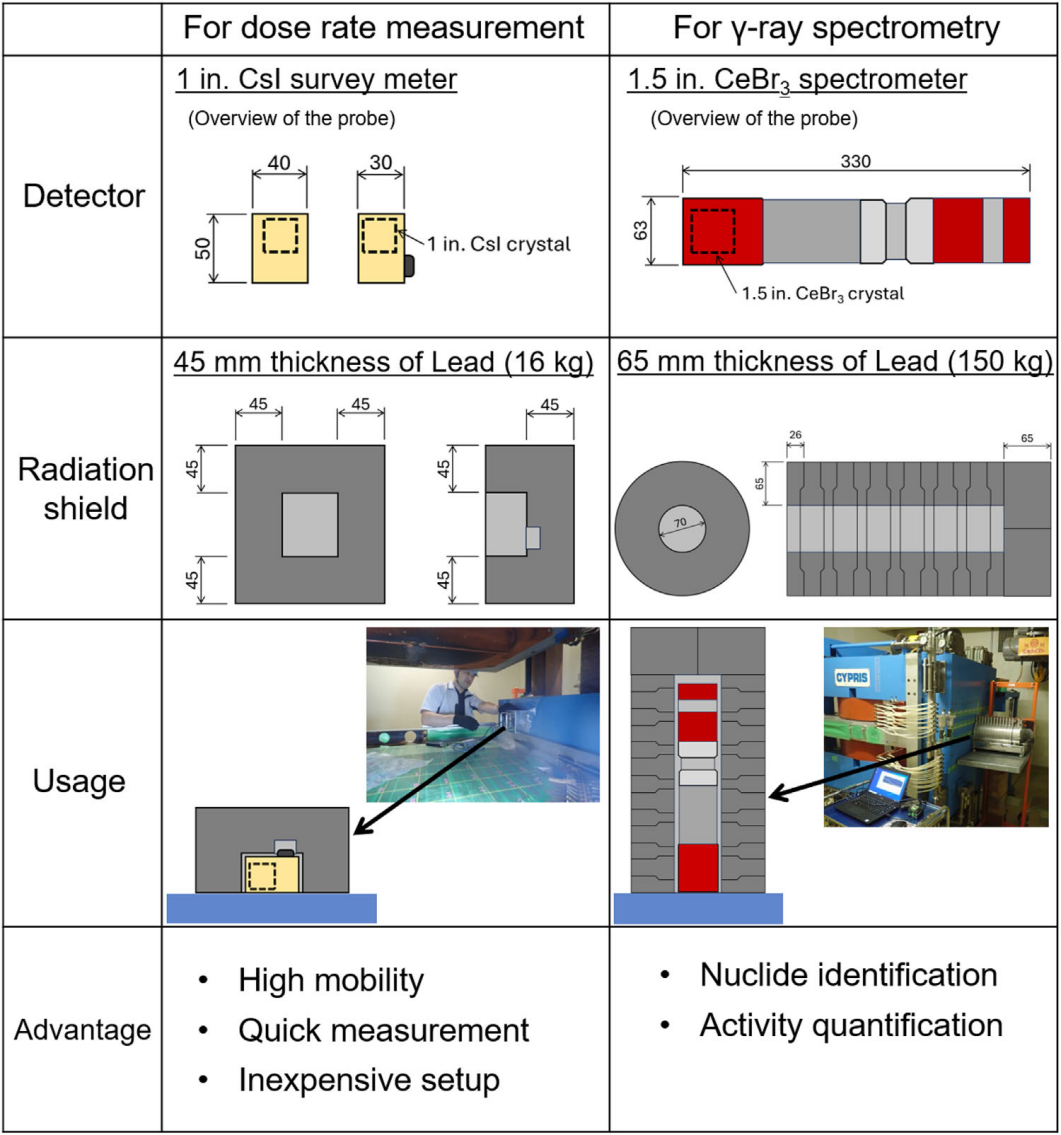
Overview and features of the experimental setups for the non‐destructive activation assessment (unit: mm).

In non‐destructive activation assessment, the outer surfaces of the object were scanned using a γ‐ray detector combined with a custom‐designed thick lead radiation shield, as shown in Figure 2. Shielding is crucial in PET cyclotron rooms, where activation elevates the ambient background radiation. The optimal lead thickness was determined experimentally; thicknesses >45 mm reduced background to <10% of the unshielded level.^3^, ^4^

Two detectors with characteristics in Figure 2 were employed. The CsI scintillation survey meter (customized T‐SP2; TAC Co. Ltd.) features a compact probe and lightweight design, offering high mobility. The CsI survey meter converts detected pulse rates into contact dose rates using an internal energy‐weighted calibration. Each measurement can be completed in a few tens of seconds, enabling rapid assessment of overall activation. Although its energy resolution is insufficient for peak‐based spectroscopy, it can still provide an indirect estimation of count rates in terms of “contact dose rate,” provided that the number of contributing radionuclides is limited. Previous studies demonstrated sufficient linearity and sensitivity of this CsI‐based method for determining specific activity in activated concrete.^2^, ^3^, ^5^, ^6^, ^7^, ^10^ In contrast, the CeBr_3_ scintillation spectrometer (CEBRS1.5 × 1.5; Mirion Technologies Co., Ltd.) offers superior energy resolution and detection efficiency, enabling direct quantification of specific activity via γ‐ray spectrometry, albeit with inferior mobility and handling ease compared with CsI.

#### 2.2.2 Determination of the conversion factor for contact dose‐rate to specific activity

As the CsI detector outputs only dose rate (no spectra) and the CeBr_3_ detector outputs spectra (no dose rate), we performed a calibration to convert contact dose rate (CsI) to specific activity (CeBr_3_) prior to the cyclotron measurements. A previous study showed that ^60^Co accounted for the majority of the residual activity in the yokes.^16^ An iron block (1320 mm × 1320 mm × 650 mm) sourced from recycled reactor materials was used as a volumetric standard (Figure S2). It had been confirmed that ^60^Co activity distributes uniformly in the block by sampling at several points. The block was repositioned with a crane to measure each face. Specific activity was derived from the 1333 keV peak area, and the detection efficiency was computed using In‐Situ Object Calibration Software (ISOCS),^21^, ^22^ assuming the actual block dimensions and uniform ^60^Co distribution. This procedure yielded a conversion factor linking the CsI contact dose rate (time constant 30 s) to the ^60^Co specific activity, providing a practical basis for the activity determination from dose‐rate measurements.

#### 2.2.3 γ‐ray measurement of the yoke and the center pole using shielded detectors

**FIGURE 3:**
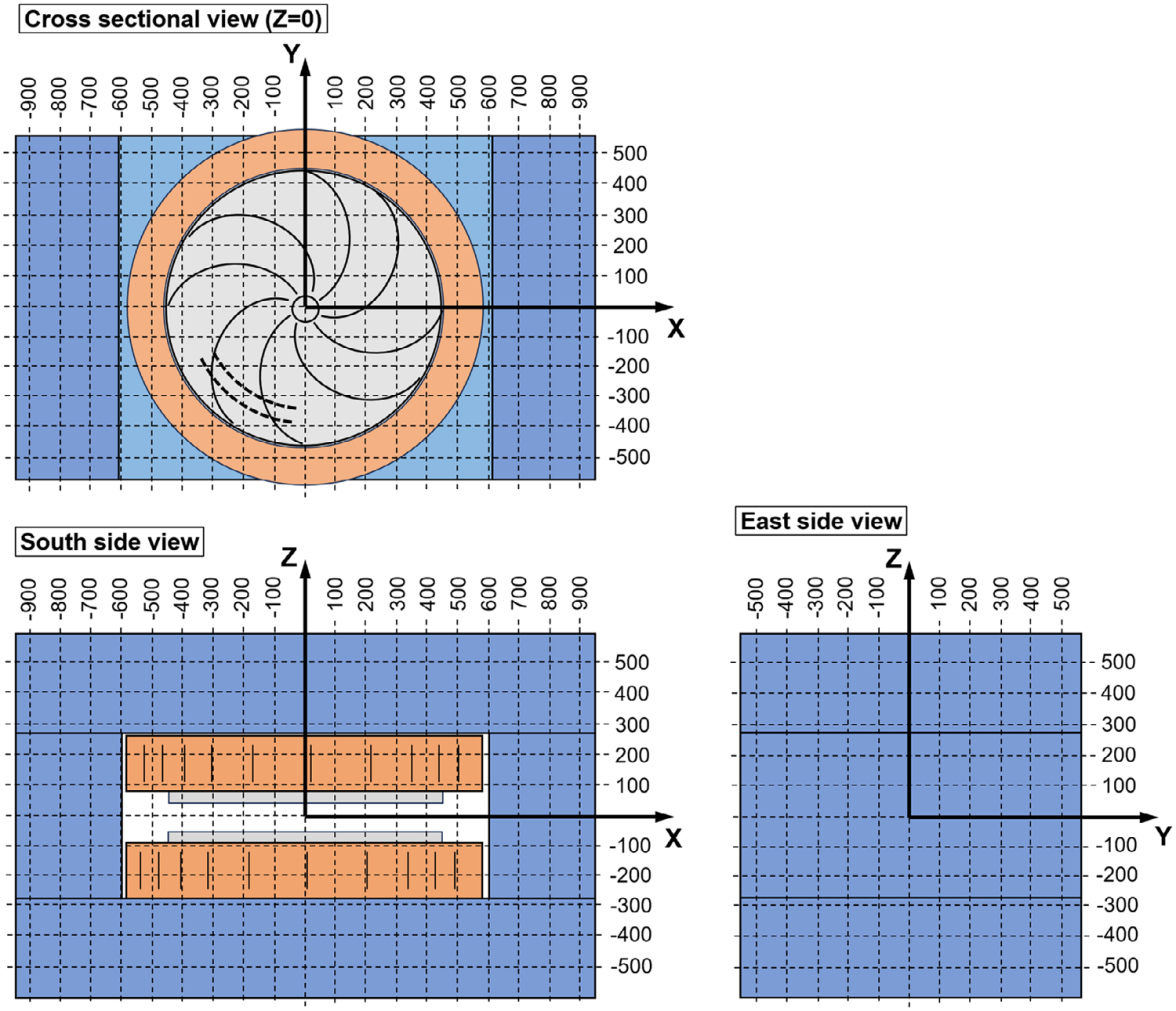
Virtual coordinate system defined on the cyclotron body at 100 mm intervals to ensure reproducibility of radiation measurements (unit: mm). Intersection points of vertical and horizontal grid lines were designated as measurement locations.

Measurements with the CsI and CeBr_3_ detectors were conducted simultaneously. To standardize positions, a custom coordinate grid with 100 mm spacing was defined on the cyclotron structure (Figure 3), balancing spatial resolution and measurement efficiency to detect localized hot spots without excessive acquisition burden. Grid lines were marked directly on the cyclotron body, and all measurement points were assigned numeric coordinates to ensure reproducibility. For the CsI, measurements were conducted manually, using the detector and shield to scan the center poles and half‐sections of the yoke. For the CeBr_3_, spectra were acquired from the four sides of the yoke (North, South, East, West) using a lift trolley for precise positioning. Standard measurements lasted 10 min; extended measurements up to ∼14 h were performed at select locations to improve counting statistics

### 2.3 Destructive activation assessment using the core‐boring method

**FIGURE 4:**
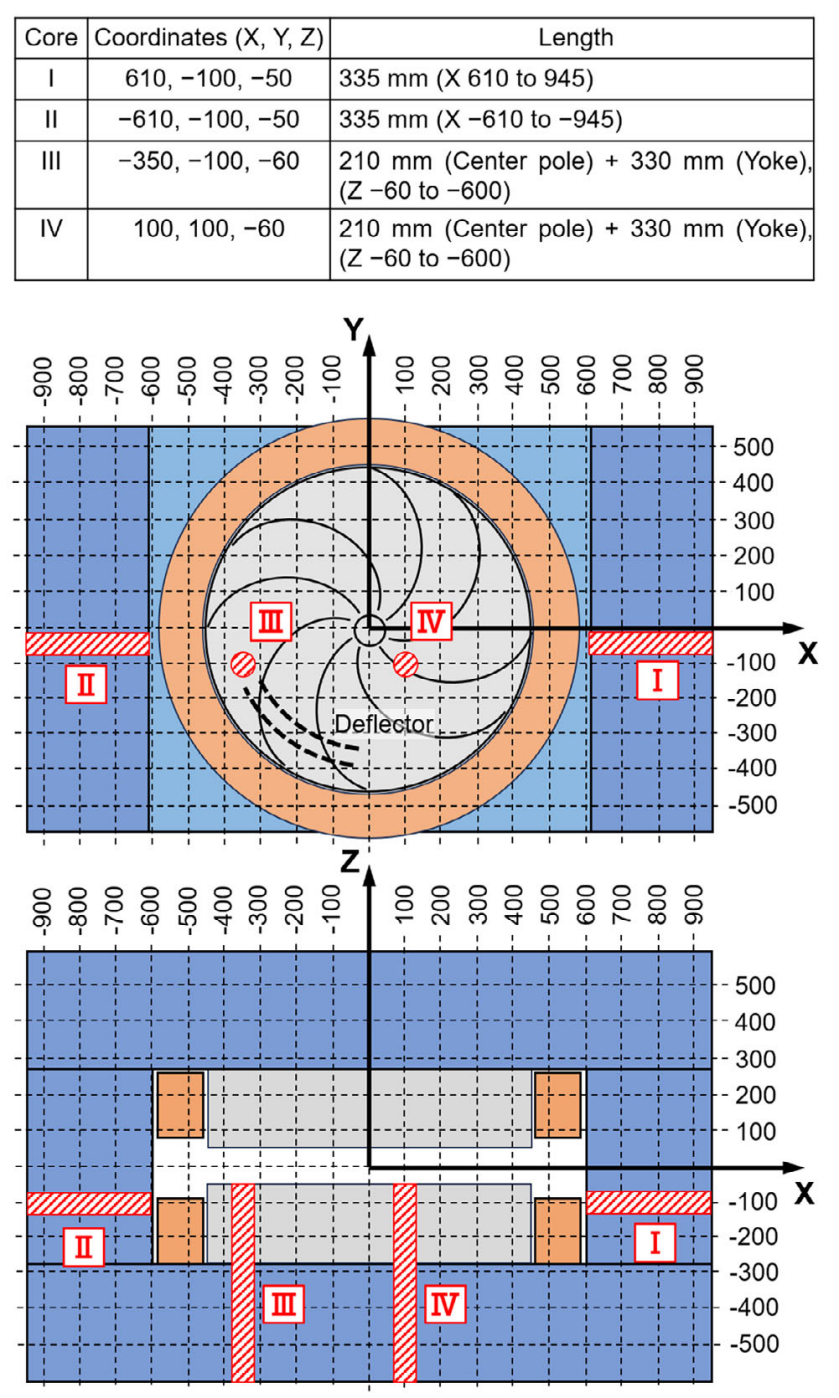
Sampling locations for destructive assessment by the core‐boring method (unit: mm).

To obtain direct measurements of internal activity, core samples were taken from four locations (Figure 4) selected to represent the overall activity distribution based on non‐destructive dose‐rate maps. Sampling was performed using a core drill with an inner diameter of 50 mm. Each core was sliced into 20 mm segments for depth‐dependent γ‐ray spectrometry.^11^ The segments were sealed in U‐8 plastic containers and analyzed with a Ge detector (Canberra, GC2518) for 20,000–30,000 s each. Detection efficiency was calculated using ISOCS.

## 3 RESULTS

### 3.1 Non‐destructive activation assessment

#### 3.1.1 Conversion factor between contact dose rate and specific activity of ^60^Co

Using shielded detectors and the iron‐block standard, we determined a conversion factor between contact dose rate and ^60^Co specific activity. The block's contact dose rate was 0.13 µSv/h and its ^60^Co specific activity was 0.27 Bq/g, yielding a factor of 2.0 (Bq/g)/(µSv/h). Thus, ^60^Co specific activity can be derived directly from CsI survey‐meter readings, facilitating activation assessment where destructive sampling is impractical.

**FIGURE 5:**
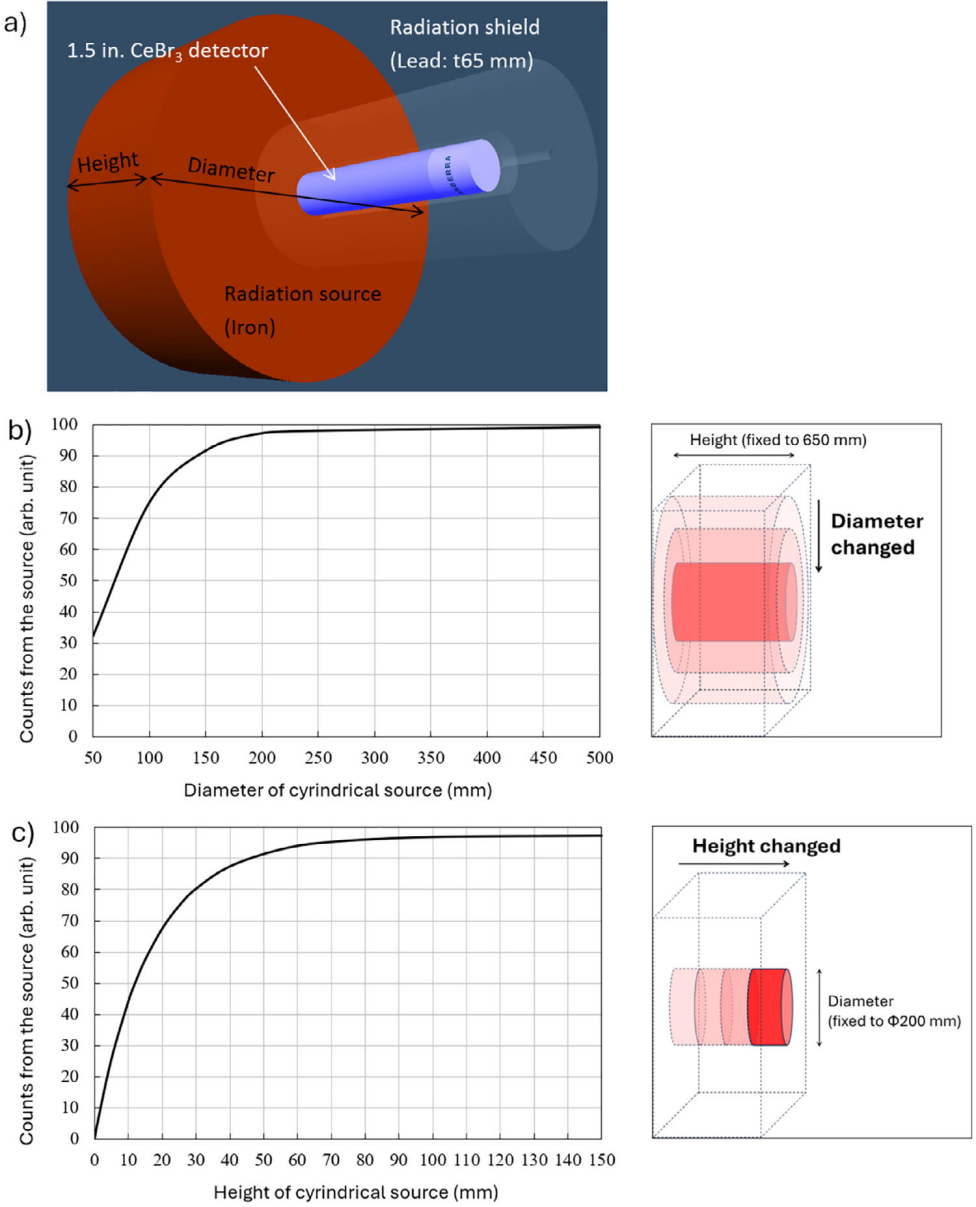
Detectable count dependence of the shielded CeBr_3_ detector with source size for 1333 keV photons emitted from an iron source, as estimated by ISOCS. (a) Hypothetical cylindrical source geometry, made of iron and containing ^60^Co uniformly. (b) Variation in detected counts when the source diameter changes with its height fixed at 650 mm. (c) Variation in detected counts when the source height changes with its diameter fixed at 200 mm. As the cylindrical source size increases, differences from the block source (1320 mm × 1320 mm × 650 mm) become indistinguishable due to self‐shielding.

ISOCS simulations (Figure 5) of CeBr_3_ photon counts at 1333 keV, using cylindrical source models with varying diameters and heights, indicated that most detectable γ‐rays originate near the detector crystal; self‐shielding produces count saturation as source size increases under the assumption of uniform activation. More than 50% of available counts arise from a diameter of ∼200 mm and a depth of ∼12 mm, and ∼80% from a depth of ∼30 mm. Accordingly, the effective detection volume of this method is localized (200 mm diameter × 30 mm depth), enabling pinpoint activity determination.

#### 3.1.2 Residual activity in the near‐surface layer of the yoke and the center pole

**FIGURE 6:**
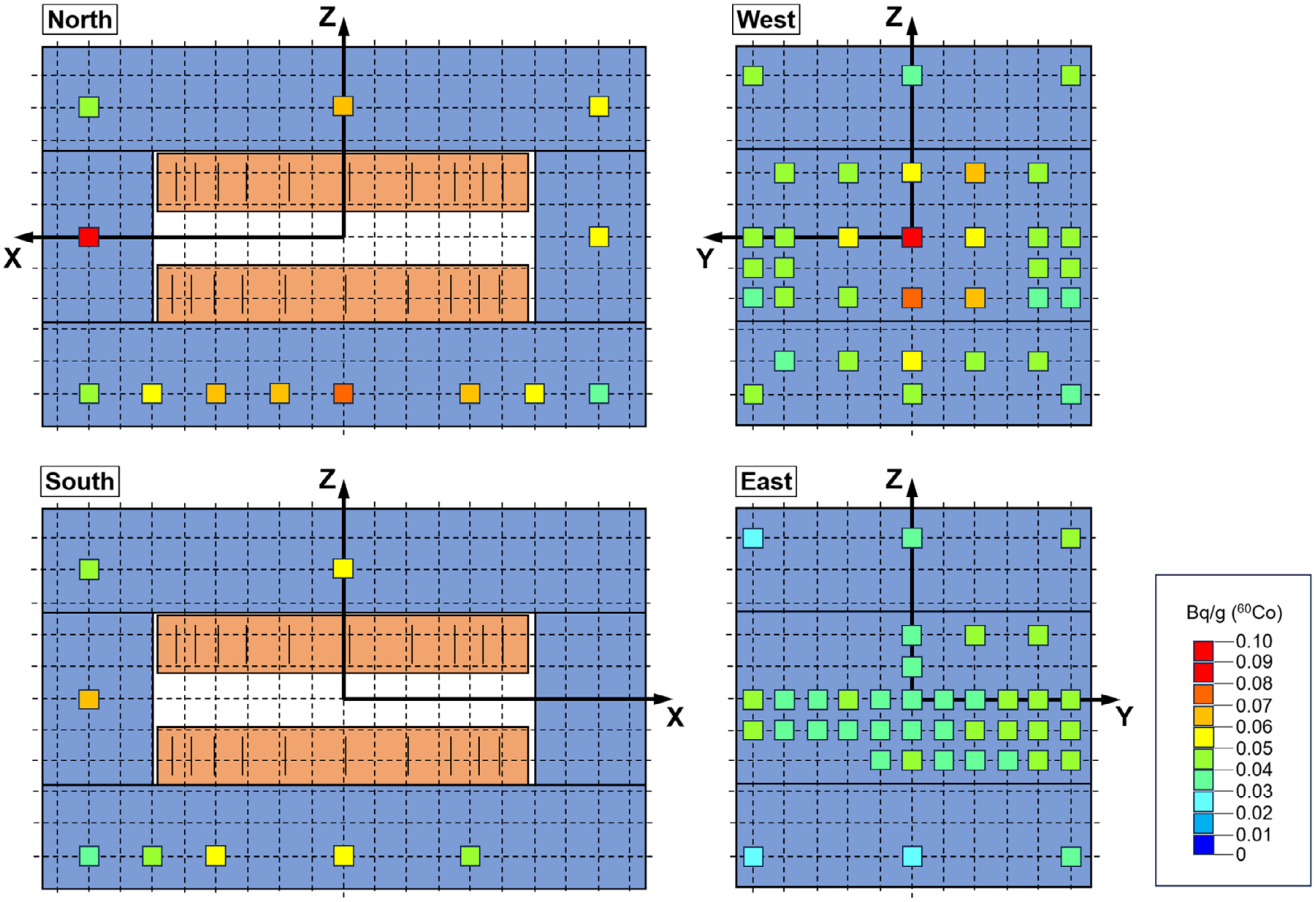
Distributions of ^60^Co residual activity for the yoke surfaces determined by the non‐destructive measurement using the CeBr_3_ detector.

Figure 6 illustrates the planar distributions of ^60^Co activity in the outer layer of the cyclotron, determined by the CeBr_3_ detector measurements. The activation levels for the four side surfaces (North, South, East, and West) were assessed. γ‐ray spectrometry detected only ^60^Co at all points, except at one location (North side, X = 800, Z = 0) near the target, where a ^54^Mn peak was observed (Figure S3 shows the γ‐ray spectrum). ^60^Co activity ranged from 0.02 to 0.1 Bq/g; nearly all points were below the 0.1 Bq/g clearance level for ^60^Co.^23^ The highest levels occurred near the deflector and target, consistent with expected beam losses.

**FIGURE 7:**
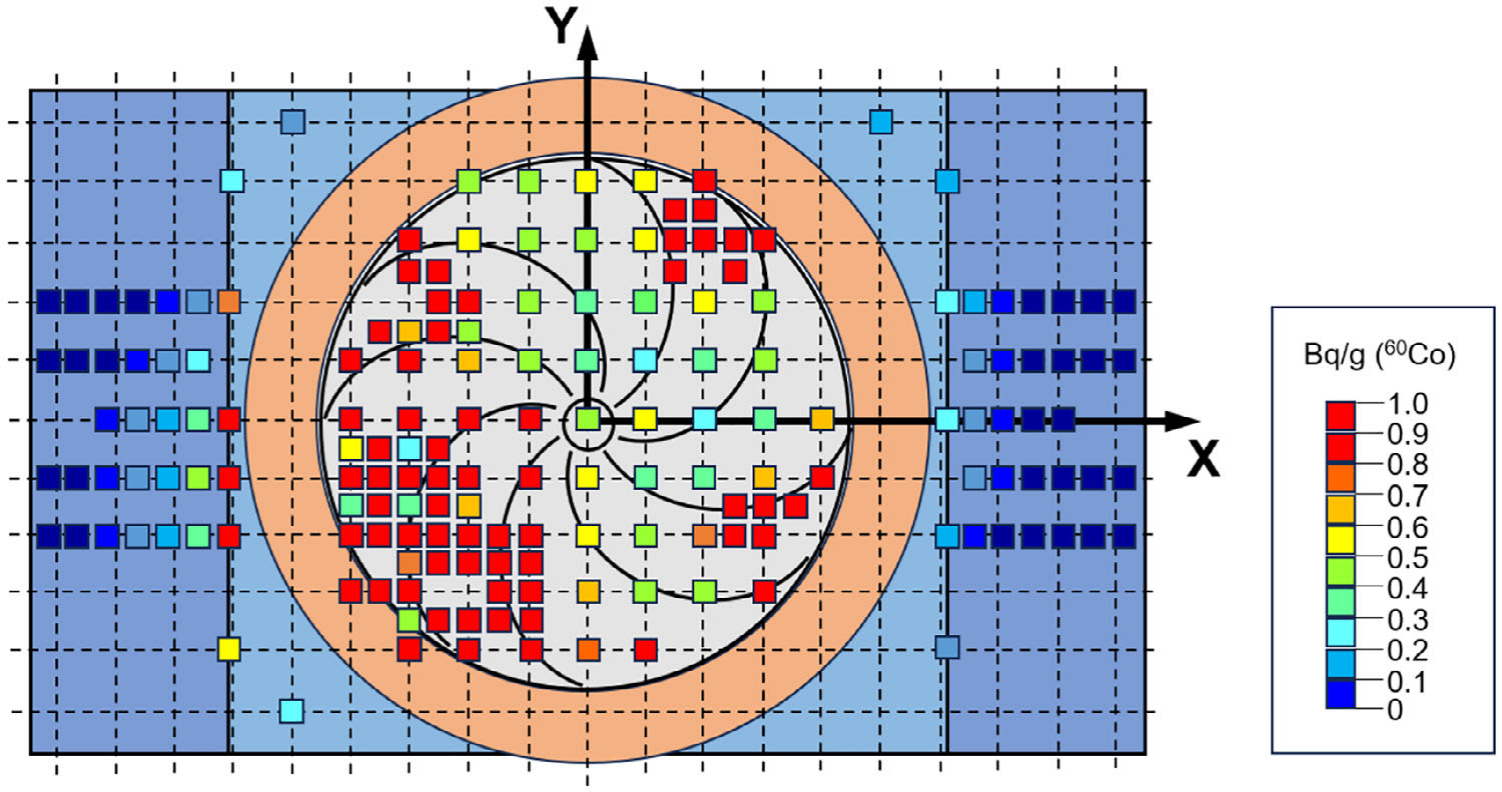
Distribution of ^60^Co residual activity for the center pole, and cross‐sections of the side yokes determined by the non‐destructive measurement using the CsI detector. During the measurement, the deflector had already been removed, but the 4 sub‐coils, which are thin coils made from oxygen‐free copper, remained on the center pole. The detector scanned over sub‐coils and consequently detected them as a pseudo‐hot spot, and these do not represent the true activation of the center pole.

Figure 7 presents the planar distribution of ^60^Co activity across the cross‐section of the side yokes and center pole, determined by the CsI measurements. In the side yokes, ^60^Co was consistently dominant (0.04–0.5 Bq/g), with activity decreasing exponentially from the central region outward. In the center pole, ^60^Co again dominated but at higher levels (0.4–6 Bq/g). Notably, activity > 1 Bq/g occurred in the region (X = –100 to –400, Y = 0 to –400), corresponding to the deflector and a magnetic‐field correction sub‐coil. Additional areas > 1 Bq/g were identified at (X = 150 to 250, Y = 250 to 400), (X = −200 to −400, Y = 100 to 300), and (X = 200 to 350, Y = −100 to −300), each coinciding with a sub‐coil location.

During the non‐destructive activation assessment, the deflector had already been removed, but the sub‐coils, which are thin coils without an iron core and made from oxygen‐free copper, the same as the deflector, remained on the center pole. Its prominent induced activity is expected to be ^60^Co too.^12^ The detector scanned over sub‐coils and consequently detected them as a pseudo‐hot spot. While this does not represent the true activation of the center pole, it suggests that hotspot detection within the effective detection volume of the non‐destructive assessment is possible even if there was significant deviation in cobalt impurities within the yoke or center pole.

### 3.2 Destructive activation assessment

**FIGURE 8:**
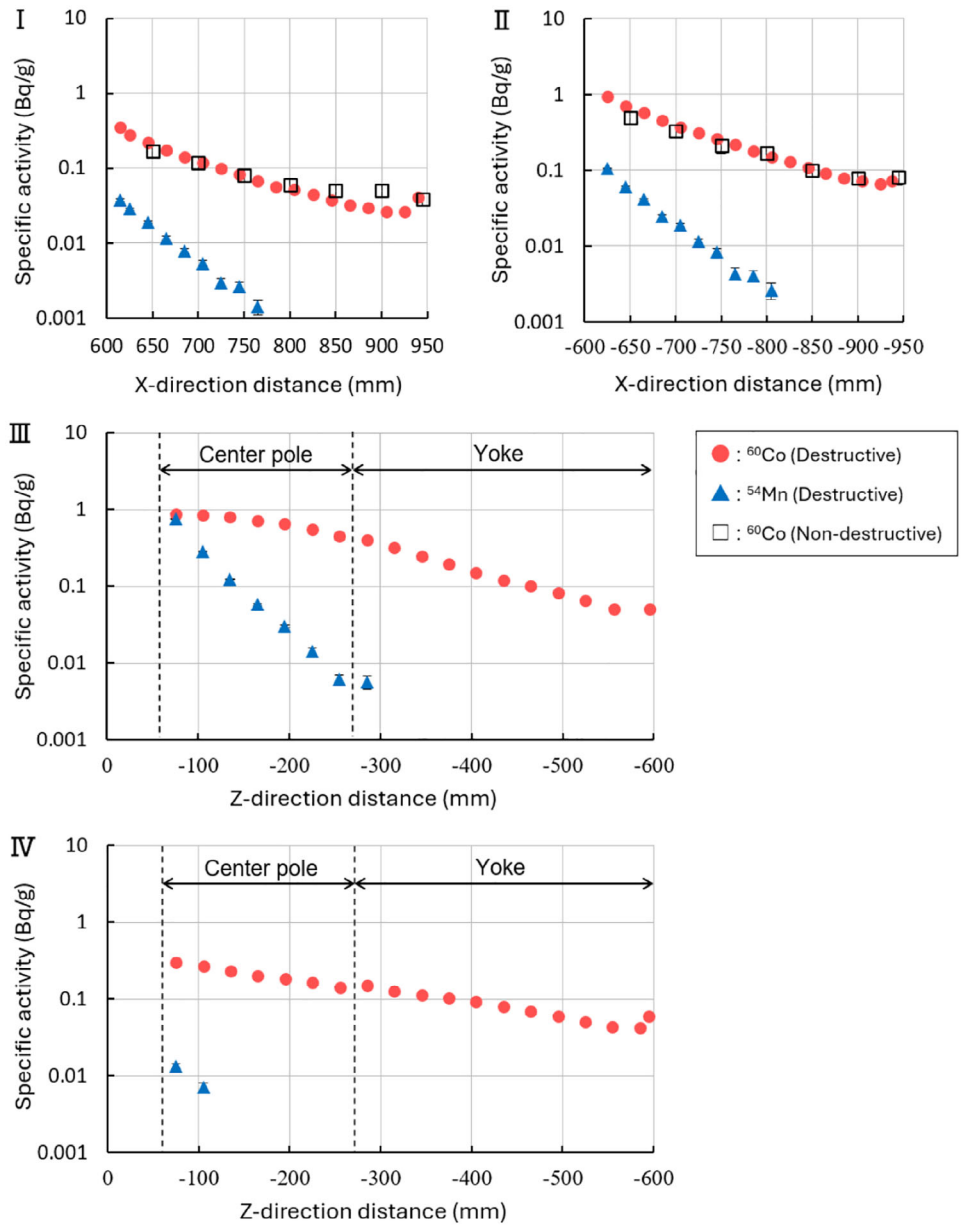
Depth distribution of residual activity in the yoke and center pole. Filled circles and triangles represent the activities of ^60^Co and ^54^Mn, respectively, determined destructively by core‐boring (Cores I–IV). Open squares represent ^60^Co activity determined non‐destructively using γ‐ray detector measurements.

Figure 8 illustrates the depth distribution of residual activity in the yokes and center pole, derived from the destructive assessment via core sampling.

Findings from Core‐I and Core‐II (Side Yokes); Consistent with non‐destructive results, ^60^Co was the principal radionuclide; ^54^Mn was one to two orders of magnitude lower. The ^60^Co depth profile derived from CsI measurements closely matched Core‐I/II results, confirming that near‐surface ^60^Co specific activity can be reliably estimated by γ‐ray detectors. The attenuation with distance: *D* (mm, from the inner surface), was approximately e^−0.008^ *^D^* for both cores. Interestingly, activity decreased with depth but increased again toward the outer surface, suggesting thermal neutrons generated by interactions with the concrete of the accelerator room penetrated inward from the exterior, inducing secondary activation.

Findings from Core‐III and Core‐IV (Center Pole and Bottom Yoke); No corresponding non‐destructive measurements were available. The center pole exhibited higher activation than the bottom yoke; maximum activities of ^60^Co and ^54^Mn were 1.0 Bq/g and 0.99 Bq/g, respectively. The bottom yoke contained negligible ^54^Mn. Outer‐surface ^60^Co levels were comparable among all four cores (0.06–0.07 Bq/g), supporting the reliability of the non‐destructive approach.

## 4 DISCUSSION

### 4.1 Contribution of ^54^Mn to non‐destructive activation assessment

The detected ^54^Mn is likely produced through fast‐neutron reactions such as ^54^Fe(n,p)^54^Mn and possibly secondary reactions involving trace elements, rather than thermal neutron capture. For the cyclotron investigated, the contribution of ^54^Mn to the dose rate in non‐destructive measurements was negligible because more than 6 years had elapsed since shutdown. However, given the 312‐day half‐life, ^54^Mn near the yoke surface is expected to exceed ^60^Co during the first year of cooling and cannot be ignored in short‐term assessments. In such cases, the ^54^Mn/^60^Co activity ratio should be derived from surface γ‐ray spectrometry using a CeBr_3_ or similar detector, and an appropriate conversion factor should be applied.^3^, ^24^ Alternatively, a conservative (safety‐conscious) estimate can be obtained by assuming all detected radiation arises from ^54^Mn, since the 1 cm dose equivalent rate coefficient of ^54^Mn (0.130) is smaller than that of ^60^Co (0.354).^25^

### 4.2 Hypothetical estimation of ^60^Co residual activity in yokes with long‐term decay

Under current Japanese regulations, all metallic components are classified as radioactive waste, leading to substantial disposal costs. Accurate activation assessment enables the identification of components that can be safely exempted from regulation and recycled. After an adequate cooling period, additional areas may fall below clearance levels, reducing total waste. Precise activation assessment is therefore essential to optimize waste management, minimize costs, and support sustainable decommissioning.

**FIGURE 9:**
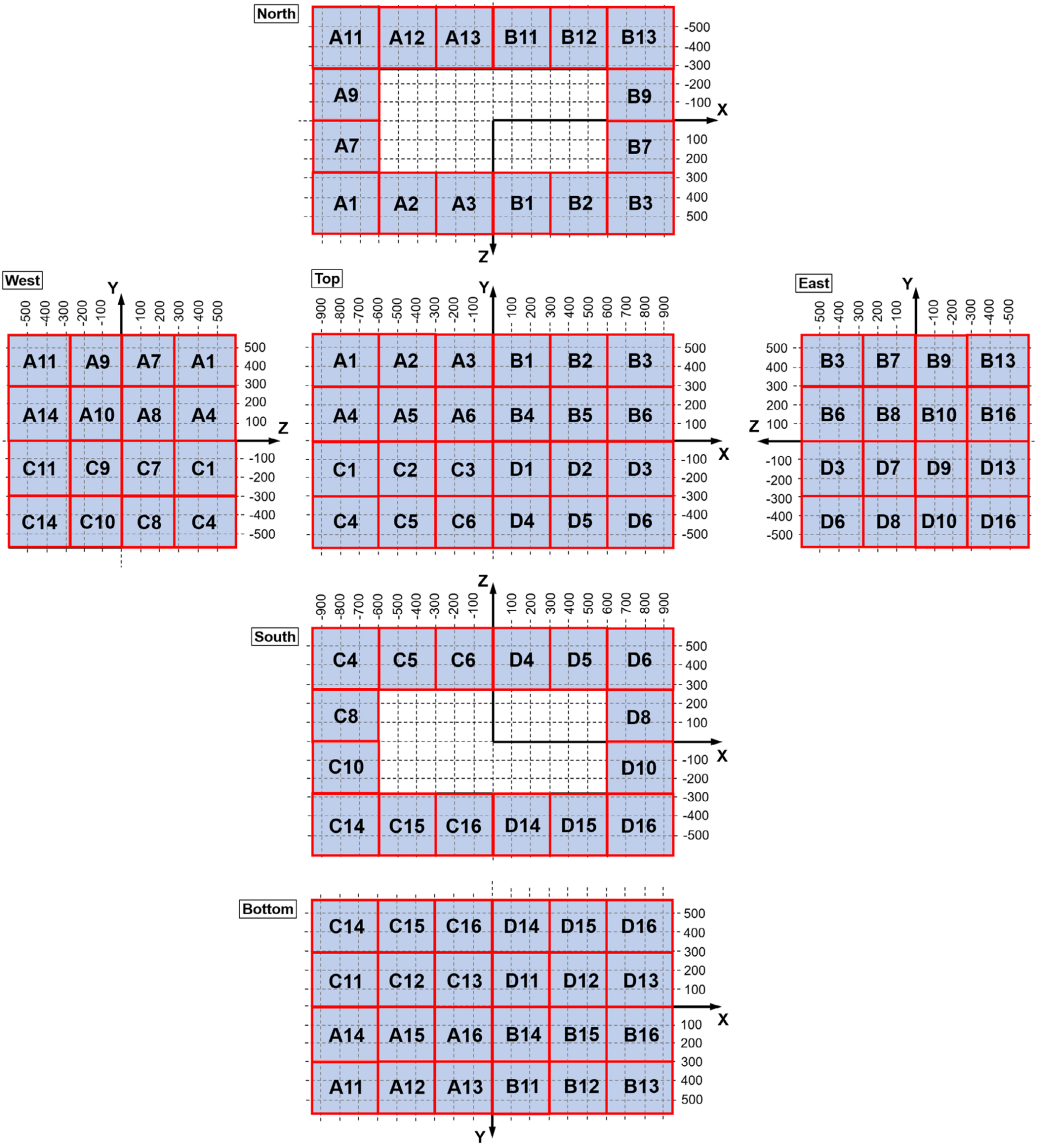
Arrangement of notional blocks dividing the yoke virtually into 64 sections for defining partial residual activity (unit: mm).

Our results confirmed that ^60^Co was the principal radionuclide contributing to metal activation in the investigated PET cyclotron. Focusing on the yokes, a major structural component, we performed a hypothetical estimation to assess how residual activity would decrease over time and how much volume could eventually meet the clearance criteria. The yokes were virtually divided into 64 notional blocks, as illustrated in Figure 9, and each block (labeled A1–D16) was treated as a representative unit for activity assignment. The block size of 300 mm was assumed, based on practical considerations for disassembly using machine tools for cutting. The estimated weight of each block ranged from 200 to 250 kg. The maximum ^6^ ^0^Co residual activity (Bq/g) was to be assigned for each block of A1 to D16 according to the following rules: 1–5.

1. The maximum ^60^Co specific activity value (*A*_max_) determined from the destructive core samplings was adopted for notional blocks that correspond to the core sampling positions. (D9 = Core‐I, C9 = Core‐II, C12 = Core‐III, D11 = Core‐IV)
2. The blocks D7, C7, C13, and D12 adjacent to D9, C9, C12, and D11 were assigned the same values of D9, C9, C12, and D11, respectively. In addition, the same values were assigned to adjacent blocks to them: A15 and A16 (assigned values from C12 and C13), and B14 and B15 (assigned values from D11 and D12).
3. For the center areas of the top yoke (blocks A5, A6, B4, B5, C2, C3, D1, and D2), symmetrical activation with the bottom yoke was assumed.
4. For the blocks B9, B10, D10, A9, A10, and C10, *A*_max_ determined by the non‐destructive approaches was adopted. The same values were also applied to their adjacent blocks, B7, B8, D8, A7, A8, and C8, respectively.
5. For all remaining blocks, *A*_max_ was assigned using the following equation:
(1) $$A_{max}=A_{surf}\cdot e^{0.008\left(D-70\right)}$$
  where *A*_surf_ represents the ^60^Co specific activity in the outer surface region determined by the non‐destructive measurement, and *D* denotes the distance from the outer surface to the opposite surface of the notional block (300 or 335 mm). Based on Core‐I/II depth profiles, ^60^Co activity was assumed constant within ∼70 mm from the surface and to increase exponentially (e^0.008^ *^D^*) beyond that depth.

**FIGURE 10:**
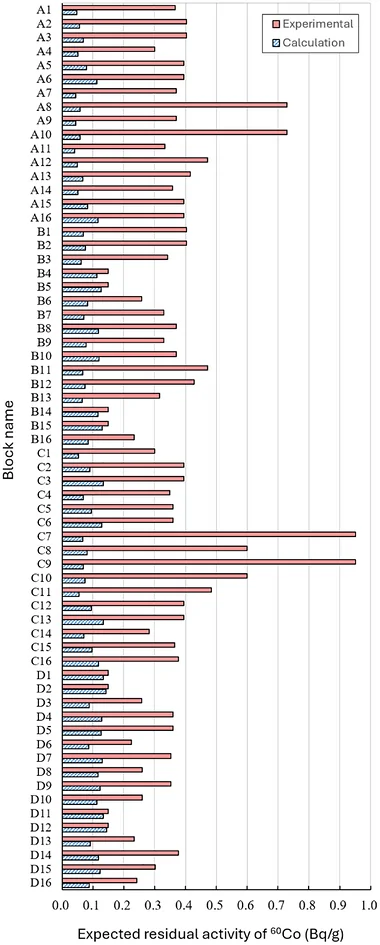
Partial ^60^Co residual activity assigned to each block of the virtually defined yoke in Figure 9. Filled bars represent values determined experimentally as Amax, and hatched bars represent values from the previous PHITS simulation.^16^

The assigned ^60^Co values for all notional blocks (Figure 10) represent conceptual residuals six years after shutdown (July 2019); none were below the 0.1 Bq/g clearance level. The figure also includes calculation results from the Particle and Heavy Ion Transport code System (PHITS),^26^ as reported previously.^16^ Although discrepancies from experimental values were observed, accurate simulation of residual activity requires realistic beam‐loss assumptions, which are difficult to define without experimental validation due to the complexity of influencing factors. Thus, experimental data provide essential benchmarks for improving the accuracy of simulation‐based assessments.

**FIGURE 11:**
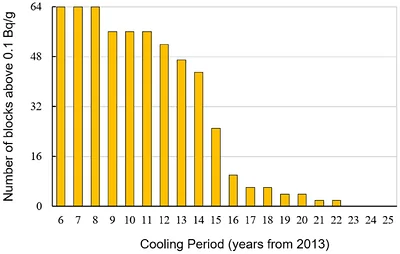
Decrease in the number of blocks with ^60^Co activity above 0.1 Bq/g due to radioactive decay during the cooling period after 2013. Sixty‐four blocks were defined as shown in Figure 9, with initial activities derived from Figure 10. The clearance level of ^60^Co (0.1 Bq/g) was used as the threshold for non‐activation.

Subsequently, a hypothetical time‐dependent radioactive decay analysis was conducted to estimate the cooling period required for the yoke to reach the clearance level. As shown in Figure 11, the decay of ^6^ ^0^Co activity was traced from 2013 (the shutdown year). The activity levels of eight blocks were estimated to be below 0.1 Bq/g, nine years after the shutdown (2022), but overall decay remained slow. After 14–16 years (2027–2029), the activity levels of 54 blocks (85%) were estimated to be below 0.1 Bq/g, indicating a rapid decline in activity levels. After 23 years (2036), the activity levels of all blocks decreased below 0.1 Bq/g. The activity levels of center poles are expected to decrease below 0.1 Bq/g after 22 years of cooling, based on the destructive core sampling results. A cooling period of approximately 23 years would be sufficient for nearly the entire cyclotron body to be below the clearance level. Assuming that the yokes and center poles consist of pure iron (density: 7.87 g/cm^3^),^27^ this corresponds to an estimated 16 tons of material that could be exempt from classification as radioactive waste. Although the results of this paper are based on a single case, the relatively uniform design and operation of PET cyclotrons suggest that the approach and findings could be extended to similar facilities.

## 5 CONCLUSIONS

An experimental activation assessment was conducted on a PET cyclotron after six years of its complete shutdown, using two complementary approaches: (1) Non‐destructive γ‐ray detector assessment and (2) Destructive core sampling.

Non‐destructive assessment: A calibration experiment was performed to convert dose rate measurements into ^60^Co activity levels using a reference iron block. This established a method for quantifying ^60^Co, the primary radionuclide responsible for residual activity, with a CsI scintillation survey meter. The effective measurement volume was localized (200 mm diameter × 30 mm depth), enabling targeted evaluation. Applied to the cyclotron, many yoke‐surface locations exhibited ^60^Co specific activity below the 0.1 Bq/g clearance level.

Destructive assessment: Core samples from the yokes and center pole provided direct internal measurements. Results confirmed ^60^Co as the dominant radionuclide and showed exponential attenuation with depth, with the highest activity near the surface. The agreement between destructive and non‐destructive results verified the reliability of γ‐ray detectors for quantitative assessment.

Finally, the long‐term decay behavior of ^60^Co activity in the yoke was predicted based on the series of assessment results to estimate the time required for the yokes to reach clearance levels after shutdown. The experimental findings also serve as benchmarks for improving the accuracy of simulation‐based activation assessments. Because PET cyclotrons are comparatively uniform across facilities, both the experimental methodology established here and the empirical results obtained can be broadly applied to accurately evaluate residual activity in metallic components such as yokes.

## CONFLICT OF INTEREST STATEMENT

This study was conducted as part of a collaborative research framework supported by Sumitomo Heavy Industries, Ltd. The authors declare that the financial support was provided solely for the purpose of facilitating experimental measurements and data analysis. The funding organization had no influence on the study design, data collection, analysis, interpretation, or manuscript preparation. The results presented in this paper do not favor any specific commercial entity and are broadly applicable for general use in the field of medical accelerator physics.

## Supporting information



Supporting Information

Supporting Information

Supporting Information

## Data Availability

The data supporting the findings of this study are not publicly available because they consist of facility‐specific measurements obtained during cyclotron decommissioning. Additional information may be available from the corresponding author upon reasonable request.
